# Influence of *UGT1A1* polymorphisms on the outcome of acute myeloid leukemia patients treated with cytarabine-base regimens

**DOI:** 10.1186/s12967-018-1579-3

**Published:** 2018-07-17

**Authors:** Peng Chen, Ke-Wei Zhu, Dao-Yu Zhang, Han Yan, Han Liu, Yan-Ling Liu, Shan Cao, Gan Zhou, Hui Zeng, Shu-Ping Chen, Xie-Lan Zhao, Jing Yang, Xiao-Ping Chen

**Affiliations:** 1 Department of Clinical Pharmacology, Xiangya Hospital, Central South University, Changsha, 410008 Hunan People’s Republic of China; 2 Institute of Clinical Pharmacology, Central South University; Hunan Key Laboratory of Pharmacogenetics, Changsha, 410078 Hunan People’s Republic of China; 3 National Clinical Research Center for Geriatric Disorders, Xiangya Hospital, Central South University, Changsha, 410008 Hunan People’s Republic of China; 4 Department of Hematology, Xiangya Hospital, Central South University, Changsha, 410008 Hunan People’s Republic of China; 5 Department of Pharmacy, The First Affiliated Hospital of Zhengzhou University, Zhengzhou, 450052 Henan People’s Republic of China

**Keywords:** Cytarabine (Ara-C), Acute myeloid leukemia (AML), UDP-glucuronosyltransferase family 1 member A1 (UGT1A1), Polymorphism

## Abstract

**Backgrounds:**

UDP-glucuronosyltransferase 1A subfamily (UGT1A) enzymes can inactivate cytarabine (Ara-C) by glucuronidation, and thus serves as candidate genes for interindividual difference in Ara-C response. UGT1A1 is a major UGT1A isoform expressed in human liver.

**Methods:**

UGT1A1*6 and *28 polymorphisms resulting in reduced UGT1A1 activity were genotyped in 726 adult acute myeloid leukemia (AML) patients treated with Ara-C based regimens. Influences of both polymorphisms on chemosensitivity and disease prognosis of the patients were evaluated.

**Results:**

After one or two courses of Ara-C based induction chemotherapy, the complete remission (CR) rate was significantly higher in patients carrying the UGT1A1*6 (77.0%) or the UGT1A1*28 (76.4%) alleles as compared with corresponding wild-type homozygotes (66.9 and 68.5%, respectively). Carriers of the UGT1A1*6 or *28 alleles showed significantly decreased risk of non-CR (OR = 0.528, 95% CI 0.379–0.737, *P *= 1.7 × 10^−4^) and better overall survival (HR = 0.787, 95% CI 0.627–0.990, *P* = 0.040) as compared with homozygotes for both polymorphisms.

**Conclusion:**

Our results suggest that UGT1A1*28 and UGT1A1*6 are associated with improved clinical outcomes in Chinese AML patients treated with Ara-C.

**Electronic supplementary material:**

The online version of this article (10.1186/s12967-018-1579-3) contains supplementary material, which is available to authorized users.

## Background

Acute myeloid leukemia (AML) is a heterogeneous hematological malignance derived from the hemopoietic progenitors with highly diverse clinical traits, molecular pathogenesis and clinical outcomes [1]. Apart from known prognostic factors including age, white blood cell (WBC) counts, complex karyotype, antecedent hematologic disease and secondary leukemia [2], accumulated evidence has shown that the presence of somatic mutations in genes such as fms-like tyrosine kinase 3 (*FLT3*), nucleophosmin 1 (*NPM1*), CCAAT enhancer binding protein alpha (*CEBPA*), KIT proto-oncogene receptor tyrosine kinase (*KIT*), tumor protein p53 (*TP53)* [3] and DNA methyltransferase 3 alpha (*DNMT3a*) [4, 5] have clinical prognostic significance. Chemotherapy with cytarabine (Ara-C) based regimen remains the major therapy for AML except for the M3 subtype, and the complete remission (CR) rate varies between 60 and 70% for the adult patients during induction therapy [6]. Half of the patients achieved CR in primarily induction chemotherapy relapsed due to the existence of minimal residual disease [7]. Overall long-term survival rate of AML ranges from 21.9 to 44% [8]. Primary or acquired chemoresistance is the major problem faced in AML treatment [9]. Therefore, identification of factors related to Ara-C-resistance and better elucidation of potential mechanisms involved in Ara-C resistance will help optimize regimens for treatment of AML and improve the clinical outcomes as well.

Ara-C is a prodrug and undergoes biotransformation into the active metabolite cytarabine triphosphate (Ara-CTP) to exert its pharmacological activity; the latter can incorporate into replicating DNA and interfere with DNA synthesis, resulting in apoptosis of cells. Three kinases are required to accomplish intracellular phosphorylation of Ara-C to the formation of Ara-CTP: the rate-limiting enzyme deoxycytidine kinase (DCK), deoxycytidine monophosphate kinase and nucleoside diphosphate kinase [10]. On the other hand, 5′-nucleotidases [11], cytidine deaminase (CDA) [12] and SAM domain and HD domain-containing protein 1 (SAMHD1) [13, 14] are the major deactivating enzymes of Ara-C that act through prevention of the formation or directly increase the degradation of the active triphosphate metabolite. Alteration in the activity of enzymes involved in Ara-C metabolism may result in a change in the proportion of its active form in the cells, and thus affects both sensitivity and toxicity of Ara-C in AML patients receiving Ara-C based chemotherapy [15]. Our previous studies have reported that polymorphisms in *DCK*, nucleoside diphosphate kinase 2 (*NME2*), ribonucleotide reductase catalytic subunit M2 (*RRM2*), and *SAMHD1* are associated with chemosensitivity to Ara-C based therapy and disease prognosis in Chinese AML patients [10, 16].

The Hedgehog (HH)/Glioma-associated Oncogene Homolog (GLI) signaling pathway that plays a role in chemotherapy resistance and cellular self-renewal is supposed to be a novel therapeutic target in AML [17]. In a phase 2 clinical trial with AML and high-risk myelodysplastic syndromes (MDS) patients, combined therapy with low dose Ara-C and a selective HH/GLI pathway inhibitor glasdegib is observed to improve overall survival (OS) as compared with Ara-C treatment alone [18]. Our recent study also showed that GLI1 expression was upregulated in bone marrow mononuclear cells from patients with refractory or relapsed AML, and GLI1 inhibition is sufficient to increase Ara-C sensitivity [19]. Furthermore, Zahreddine and colleagues demonstrated that UDP-glucuronosyltransferase 1A subfamily (UGT1A) enzymes can inactivate Ara-C through glucuronidation in leukemia cells, and GLI1 is involved in Ara-c resistance through enhancing the stability of UGT1A enzymes [20]. However, there is no report on associations of genetic polymorphisms in *GLI1* and the *UGT1A* subfamily with Ara-C presently.

The human UGT1A subfamily enzymes are encoded by the *UGT1A* gene locus on Chromosome 2q37.1 by alternative splicing. Nine functional proteins (UGT1A1, 1A3, 1A4, 1A5, 1A6, 1A7, 1A8, 1A9, and 1A10) were translated by the locus. All the isoforms share 4 common exons from exon 2 to exon 5, but have unique exon 1 and individual promoter pairs in turn of 1A8, 1A10, 1A9, 1A7, 1A6, 1A5, 1A4, 1A3, 1A1 in the *UGT1*A pre-mRNA [21]. The *UGT1A1* exon 1/promoter is the nearest one to the common exon 2. UGT1A1 plays important roles in the clearance and metabolism of many endogenous or exogenous compounds such as irinotecan [22]. Two functional single nucleotide polymorphisms (SNPs) in *UGT1A1*, i.e. UGT1A1*28 (rs8175347) and UGT1A1*6 (rs4148323) are reported. The UGT1A1*28 polymorphism that results in TA repeating number alteration in the TATA box in the promoter can decrease the *UGT1A1* expression and accounts for increased risk for irinotecan-induced neutropenia [23, 24] and myelosuppression [25, 26]. In 2005, the USA Food and Drug Administration (FDA) warned that patients with UGT1A1*28/*28 genotype are at increased risk for neutropenia when irinotecan is used, and a lower starting dose of irinotecan was recommended for UGT1A1*28/*28 homozygotes [27]. UGT1A1*6 is a missense variants (Gly71Arg) in *UGT1A1* exon 1 that leads to decreased UGT1A1 enzyme function [28] and is also a risk factor for irinotecan toxicity. UGT1A1*6 variant is mainly observed in the Asians with allele frequency ranges in 15–30%. In 2008, the Ministry of Health, Labour, and Welfare of Japan also warned increased risk of severe irinotecan-related neutropenia in Japanese patients carrying the UGT1A1*6 or *28 allele, and approved diagnostic test for *UGT1A1* genotypes [29]. As UGT1A is involved in Ara-C detoxification, we hypothesized that the functional polymorphisms in *UGT1A1* may affect chemosensitivity to Ara-C based therapies in AML patients through influence Ara-C metabolism, which could eventually improve responses to Ara-C and AML prognosis.

In this study, we investigated the impact of UGT1A1*28 and *6 variants on CR rate after induction chemotherapy, treatment-related mortality (TRM), OS and event-free survival (EFS) in 726 Chinese AML patients treated with Ara-C.

## Methods

### Study design and patient population

In this study, a total of 726 patients were enrolled at Xiangya Hospital, Central South University between May 2009 and Feb 2017. This study was approved by the Ethics Committee of Institute of Clinical Pharmacology of Central South University (No. CTXY-120025-2) and conducted in compliance with the provisions of the Declaration of Helsinki (Chinese Clinical Trial Register: ChiCTR-PPC-14005297). All patients provided written informed consent that explicitly included genetic information sharing with qualified investigators before enrollment.

The patients > 14 years of old of age, diagnosed with de novo AML or secondary AML (according to the WHO criteria), and were planned to be treated with Ara-C based induction chemotherapy were enrolled. Those diagnosed as acute promyelocytic leukemia (M3 AML), therapy-related AML (T-AML), acute mixed lineage leukemia, or accompanied by other serious diseases were excluded from this study. For the induction therapy, patients received a standard-dose of Ara-C (100–200 mg/m^2^ continuous infusion × 7 days) in combination with one of the anthracyclines (daunorubicin 45–90 mg/m^2^, or idarubicin 10–20 mg/m^2^, or aclarubicin 20 mg/m^2^, or pirarubicin 30 mg/m^2^, or mitoxantrone 8–16 mg/m^2^ × 3 day). Granulocyte colony-stimulating factor (G-CSF) were used for some patients for the prevention or treatment of myelosuppression. Some elderly patients received low-intensity therapy regimens included low-dose Ara-C (10–20 mg/m^2^ × 14 days) were also enrolled. Once CR was achieved, the patients were treated with sequential consolidation therapy consisting of Ara-C and anthracyclines or hematopoietic stem cell transplantation (HSCT). Clinical information of the patients was collected from the medical records, regular outpatient review, and trimonthly telephone following-up. All clinical events were recorded at least once per 3 months and the follow-up ended on Nov 31st, 2017. A flow diagram of the process for participant recruitment was illustrated in Additional file 5: Fig. S2.

### Response criteria and evaluation of end points

The primary endpoint was drug response, which was categorized as CR or non-CR after the second cycle of the induction chemotherapy according to the criteria of International Working Group AML criteria [30]. The criteria of CR was defined as follows: less than 5% blasts and no blasts with Auer rods in the bone marrow, no persistence of extramedullary disease, absolute neutrophil count of > 1×10^9^/l and platelets of > 100 × 10^9^/l, and independent of transfusion. Participants with other treatment outcomes including partial remission, non-remission and TRM after two courses of induction chemotherapy were classified as non-CR group. As mortality tended to be decreased sharply in 4 weeks after the initiation of induction therapy in de novo AML [31], TRM was defined as death within 28 days after initiation of induction therapy. OS was calculated from the date of diagnosis until death from any cause, and EFS was calculated since the date of CR until the date of relapse or death from any cause. Patients who underwent HSCT after achievement of CR were censored at the date of HSCT for OS and EFS. For those patients living or with no evidence of relapse by the end of the study follow-up, time for OS or EFS was censored on the date of the patient’s last follow-up [30].

### Genotyping of UGT1A1*6 and *28

Peripheral venous blood samples were collected into EDTA-anticoagulate tubes. Genomic DNA was extracted using a E.Z.N.A.^®^ SQ Blood DNA Kit II (Omega Bio-Tek, USA) according to the manufacturer’s instructions and stored at − 80 °C until use. Genotyping of *UGT1A1* polymorphisms was carried out by polymerase chain reaction (PCR) and pyrosequencing as described previously [32]. Briefly, The DNA fragments flanking the UGT1A1*28 or *6 polymorphisms were amplified using Mastercycler (Eppendorf, Germany) in a final reaction volume of 50 µl, which contained 2 µl DNA, 5 µl PCR buffer, 1.5 µl dNTP, 0.5 ul DNA polymerase, 0.05 nM of each amplification primer (Additional file 1: Table S1), and 40 μl sterile double-distilled water. The thermal cycling of PCR was used as follows: degeneration at 94 °C for 5 min; 35 cycles of 94 °C for 30 s, 58 °C or 66 °C for 30 s respectively, and 72 °C for 30 s; and a final extension 72 °C for 7 min. The PCR products were verified by agarose electrophoresis, followed by pyrosequencing on the PyroMark Q24 Advanced platform (Qiagen, Germany) using PyroMark Reagents (Qiagen, Germany) with the pyrosequencing primer (Additional file 1: Table S1). Genotyping results of each SNP were verified in 5% samples selected randomly using Sanger sequencing.

### Statistical analysis

Comparisons of continuous data between genotype groups were performed by independent Student’s T test or Mann–Whitney U test. Fisher’s exact test, Continuity correction or Pearson Chi Square test were applied to assess differences in chemotherapy response, toxicity and other clinical information between the genotypes. Logistic regression analysis was carried out to estimate the relative risk of non-CR adjusted for age, risk stratification, and WBC count. The Kaplan–Meier curves were depicted and log-rank test was performed to determine the differences in OS and EFS between/among genotype groups. Hazard ratios for OS and EFS were estimated by Cox proportional hazards model adjusting for potential confounding covariates (including risk stratification, WBC count, and age). All analyses were performed using the SPSS software version 18.0 (IBM Corporation, USA), and the level of statistical significance was defined as *P* < 0.05 in a two-sided test. The HaploView 4.2 software was used to analyze Hardy–Weinberg equilibrium (HWE) of the genotypes and linkage disequilibrium between the SNPs.

## Results

### Patient characteristics and follow-up

A total of 726 AML patients were eligible and adopted in the study. The baseline characteristics of the patients were summarized in Table 1. The mean age of the patients was 41 years, and 78 patients (10.7%) were aged 60 or more. M2 (51.2%) was the most frequent French–American–British (FAB) subtype for the patients, followed by M4 (20.5%) and M5 (20.1%). Risk stratification based on cytogenetic and molecular abnormalities was available for 622 patients: 152 low risk, 330 intermediate risk, and 140 high risk. *FLT3* mutation status, karyotypes and the first induction therapy regimens were shown in Additional file 2: Table S2. During the first induction therapy, 692 patients received standard-dose Ara-C with anthracycline or mitoxantrone regimens, and 34 (4.7%) patients received low-intensity regimens of decitabine and subcutaneous Ara-C. G-CSF was given to 137 (18.9%) patients simultaneously to avoid myelosupression (Additional file 2: Table S2).

**Table 1:** Clinical features of AML patients according to *UGT1A1* genotypes

Clinical features	Total (n = 726)	UGT1A1*28			UGT1A1*6		
		*1/*1 (n = 579)	*28/− (n = 147)	*P* value	*1/*1 (n = 493)	*6/− (n = 233)	*P* value
Age, years	41 ± 14	41 ± 14	41 ± 15	0.816	41 ± 14	42 ± 14	0.799
Male sex, n (%)	400 (55.1)	317 (54.7)	83 (56.5)	0.709	280 (56.8)	120 (51.5)	0.181
Smoking, n (%)	171(23.6)	141 (24.4)	30 (20.4)	0.314	125 (25.4)	46 (19.7)	0.096
Drinking, n (%)	108 (14.9)	91 (15.7)	17 (11.6)	0.206	69 (14.0)	39 (16.7)	0.332
FAB classification, n (%)							
M0	3 (0.4)	2 (0.3)	1 (0.7)	0.918	1 (0.2)	2 (0.9)	0.204
M1	38 (5.2)	10 (5.2)	11 (7.5)		33 (6.7)	8 (3.4)	
M2	372 (51.2)	298 (51.5)	74 (50.3)		258 (52.3)	114 (48.9)	
M4	149 (20.5)	118 (20.4)	28 (19.0)		94 (19.1)	52 (23.3)	
M5	146 (20.1)	117 (20.2)	29 (19.7)		97 (19.7)	49 (21.0)	
M6	17 (2.3)	13 (2.2)	4 (2.7)		9 (1.8)	8 (3.4)	
M7	1 (0.1)	1 (0.2)	–		1 (0.2)	–	
AML type, n (%)							
De novo AML	692 (95.3)	551 (95.2)	141 (95.9)	0.699	469 (95.1)	223 (95.7)	0.732
Secondary AML	34 (4.7)	28 (4.8)	6 (4.1)		24 (4.9)	10 (4.7)	
Parameters at diagnosis							
WBC count, × 10^9^/l	40.07 ± 62.15	39.54 ± 62.53	42.17 ± 60.80	0.650	40.70 ± 59.07	38.72 ± 68.37	0.691
RBC count, × 10^12^/l	2.33 ± 1.64	2.26 ± 0.77	2.60 ± 3.33	0.232	2.39 ± 1.93	2.21 ± 0.73	0.173
Hemoglobin, g/l	73.98 ± 21.68	73.64 ± 21.86	75.36 ± 20.99	0.395	74.61 ± 22.25	72.66 ± 20.44	0.261
Platelets count, × 10^9^/l	55.41 ± 64.40	54.70 ± 60.73	58.22 ± 77.45	0.559	53.90 ± 61.17	58.62 ± 70.80	0.360
Neutrophil count, × 10^9^/l	13.44 ± 33.00	13.51 ± 34.56	13.14 ± 26.04	0.905	13.98 ± 32.47	12.29 ± 34.23	0.525
LDH, U/l	564.6 ± 725.2	561.2 ± 771.9	578.2 ± 501.1	0.806	566.4 ± 758.5	560.9 ± 651.7	0.926
Bone marrow blasts, %	65.37 ± 20.88	64.94 ± 20.81	67.02 ± 21.12	0.291	65.98 ± 21.21	64.08 ± 20.15	0.267
Risk stratifications, n (%)							
Low risk	152 (20.9)	115 (19.9)	37 (25.2)	0.553	98 (19.9)	54 (23.2)	0.142
Intermediate risk	330 (45.5)	267 (46.1)	63 (42.9)		230 (46.7)	100 (42.9)	
High risk	140 (19.3)	114 (19.7)	26 (17.7)		102 (20.7)	38 (16.3)	
Unknown	104 (14.3)	83(14.3)	21 (14.3)		63 (12.8)	41 (17.6)	
HSCT, n (%)	124 (17.1)	94 (16.2)	30 (20.4)	0.230	89 (18.1)	35 (15.0)	0.311

*28/− or *6/− represents mutant homozygous and heterozygous of corresponding loci on *UGT1A1*

Risk stratification based on NCCN guidelines version 1.2015 acute myeloid leukemia

*FAB Classification* French–Britain–American Classification, *HSCT* hematopoietic stem cell transplantation, *LDH* lactate dehydrogenase, *RBC* red blood cell, *WBC* white blood cell

*P* value was based on χ^2^ test for categorical variables and student’s *t* test for continuous variables

The overall CR rate after the first cycle of induction therapy was 40.2% (288/716). Assessment of response to chemotherapy after two courses of Ara-C based induction chemotherapy was available for 697 patients, and 489 (70.1%) patients achieved CR after 2 cycles of induction therapy. A total of 567 patients achieved CR ultimately after induction therapy. The mean and median follow-up periods for the 726 patients were 667 and 434 days, respectively (range 25–2903 days). The median EFS and OS values for the patients were 488 days and 607 days, respectively. During the follow-up, 232 (40.9%, 232/567) patients relapsed after achievement of CR, and 373 (51.4%, 373/726) patients died by the end of the follow-up period.

UGT1A1*6 and UGT1A1*28 polymorphisms were genotyped in the patient cohort. For the UGT1A1*6 polymorphism, genotype frequencies for the *1/*1, *1/*6, and *6/*6 were 493 (67.9%), 209 (28.8%), and 24 (3.3%), respectively. For the UGT1A1*28 polymorphism, genotype frequencies for the *1/*1, *1/*28 and *28/*28 were 79.8% (579 patients), 18.2% (132 patients), and 2.0% (15 patients), respectively. The genotype distribution was in accordance with HWE for the UGT1A1*6 polymorphism (*P *= 0.819) but not the UGT1A1*28 polymorphism (*P *= 0.048). No obvious linkage disequilibrium (LD) between the two polymorphisms was observed (r^2^ = 0.027). Overall, no significant difference in baseline characteristics was observed between patients carrying the wild-type and mutant *UGT1A1* genotypes (Table 1).

### Associations between *UGT1A1* polymorphisms and response to chemotherapy

Comparisons of response to two courses of induction therapy among the *UGT1A1* genotypes were shown in Table 2. Patients carrying the UGT1A1*6 allele (*1/*6 + *6/*6 genotypes) showed significantly higher CR rate after two cycles of induction therapy compared to those carrying the *1/*1 genotype (77.0% vs 66.9%, *P *= 0.007). Similarly, carriers of the UGT1A1*28 allele (*1/*28 + *28/*28 genotypes) also showed marginally significantly increased CR rate than the *1/*1 homozygotes (76.4% vs 68.5%, *P *= 0.068). When combined genotypes for both polymorphisms were considered, wildtype homozygotes for both polymorphisms showed the lowest CR rate (63.8%), carries of the UTG1A1*6 alone, UTG1A1*28 alone, and each of the variant allele (*6 or *28) showed a CR rate of 77.3, 76.8, and 76.9%, respectively. Results of unconditional logistic regression showed that carriers of UTG1A1*6 showed significantly reduced risk of non-CR (OR = 0.604; 95% CI 0.419–0.869, *P *= 0.007), and carriers of the UTG1A1*28 showed marginally decreased risk of non-CR (OR = 0.673, 95% CI 0.440–1.029, *P *= 0.068) as compared with corresponding wildtype genotype. As the two polymorphisms showed low LD in our patients, association of the combined genotypes with non-CR risk was also analyzed. Significantly decreased risk of non-CR was observed in patients carrying the UGT1A1*6 or *28 alleles as compared with homozygotes for both polymorphisms (OR = 0.528, 95% CI 0.379–0.737, *P *= 1.7 × 10^−4^, Table 1). Patients with the UGT1A1*28/− or *6/− also showed decreased risk of non-CR after the first induction therapy (OR = 0.672, 95% CI 0.498–0.907, *P *= 0.009, Additional file 3: Table S3). No significant difference in TRM among the UGT1A1*6 and UGT1A1*28 genotypes was observed (Additional file 4: Table S4).

**Table 2:** Comparison of CR rate among *UGT1A1* genotypes after 2 courses of Ara-C based chemotherapy

SNP	Genotype	Total, n	CR, n (%)	Non-CR, n (%)	OR (95% CI)	*P* value
UGT1A1*6	*1/*1	471	315 (66.9)	156 (33.1)	1.00 (reference)	
	*1/*6	202	155 (76.7)	47 (23.3)	0.612 (0.419–0.894)	0.011
	*6/*6	24	19 (79.2)	5 (20.8)	0.531 (0.195–1.450)	0.353
	*6/−	226	174 (77.0)	52 (23.0)	0.604 (0.419–0.869)	0.007
UGT1A1*28	*1/*1	553	379 (68.5)	174 (31.5)	1.00 (reference)	
	*1/*28	130	97 (74.6)	33 (25.4)	0.741 (0.480–1.144)	0.176
	*28/*28	14	13 (92.9)	1 (7.1)	0.168 (0.022–1.291)	0.086
	*28/−	144	110 (76.4)	34 (23.6)	0.673 (0.440–1.029)	0.068
Combined genotypes	*1/*1 for both loci	359	229 (63.8)	130 (36.2)	1.00 (reference)	
	*6/− alone	194	150 (77.3)	44 (22.7)	0.517 (0.347–0.770)	0.001
	*28/− alone	112	86 (76.8)	26 (23.2)	0.533 (0.327–0.868)	0.011
	*6/− and *28/−	32	24 (75.0)	8 (25.0)	0.587 (0.256–1.345)	0.208
	*6/− or *28/−	338	260 (76.9)	78 (23.1)	0.528 (0.379–0.737)	1.7 × 10^−4^

When adjusted by clinical factors, results of multivariate analysis showed that AML risk stratification, pretreatment WBC count, *UGT1A1* genotypes based on *6 and *28 concomitantly, and age showed significant association with non-CR risk after two-courses of induction therapy (Table 3). In details, carriers of the UGT1A1*6 or *28 allele showed decreased risk of non-CR (OR = 0.547, 95% CI 0.375–0.797, *P *= 0.002); low risk stratification and high risk stratification showed decreased (OR = 0.457, 95% CI 0.269–0.776, *P *= 0.004) and increased (OR = 2.045, 95% CI 1.331–3.140, *P *= 0.001) risk of non-CR, respectively, as compared with intermediate risk stratification; pretreatment WBC count and age was associated with increased risk of non-CR (WBC count: OR = 1.004, 95% CI 1.001–1.007, *P *= 0.008 for; Age: OR = 1.014, 95% CI 1.001–1.028, *P *= 0.036). Association between UGT1A1*6 or *28 carrying status, risk stratification and pretreatment WBC count were also associated with risk of non-CR for the first induction therapy (Table 3).

**Table 3:** *UGT1A1* polymorphisms and clinical variables of multivariate analysis for non-CR risk in AML

Variables in the model	Non-CR risk after two courses of induction		Non-CR risk after the first course of induction	
	OR (95% CI)	*P* value	OR (95% CI)	*P* value
UGT1A1 *28/− or *6/−	0.547 (0.375–0.797)	0.002	0.622 (0.446–0.868)	0.005
Risk stratification groups		2.5 × 10^−6^		0.021
Low vs intermediate	0.457 (0.269–0.776)	0.004	0.678 (0.455–1.011)	0.056
High vs intermediate	2.045 (1.331–3.140)	0.001	1.365 (0.880–2.116)	0.164
WBC count, × 10^9^/l	1.004 (1.001–1.007)	0.008	1.005 (1.001–1.008)	0.011
Age, years	1.014 (1.001–1.028)	0.036	1.005 (0.993–1.017)	0.448

*WBC* white blood cell

### Association between *UGT1A1* polymorphisms and AML prognosis

**Fig. 1:**
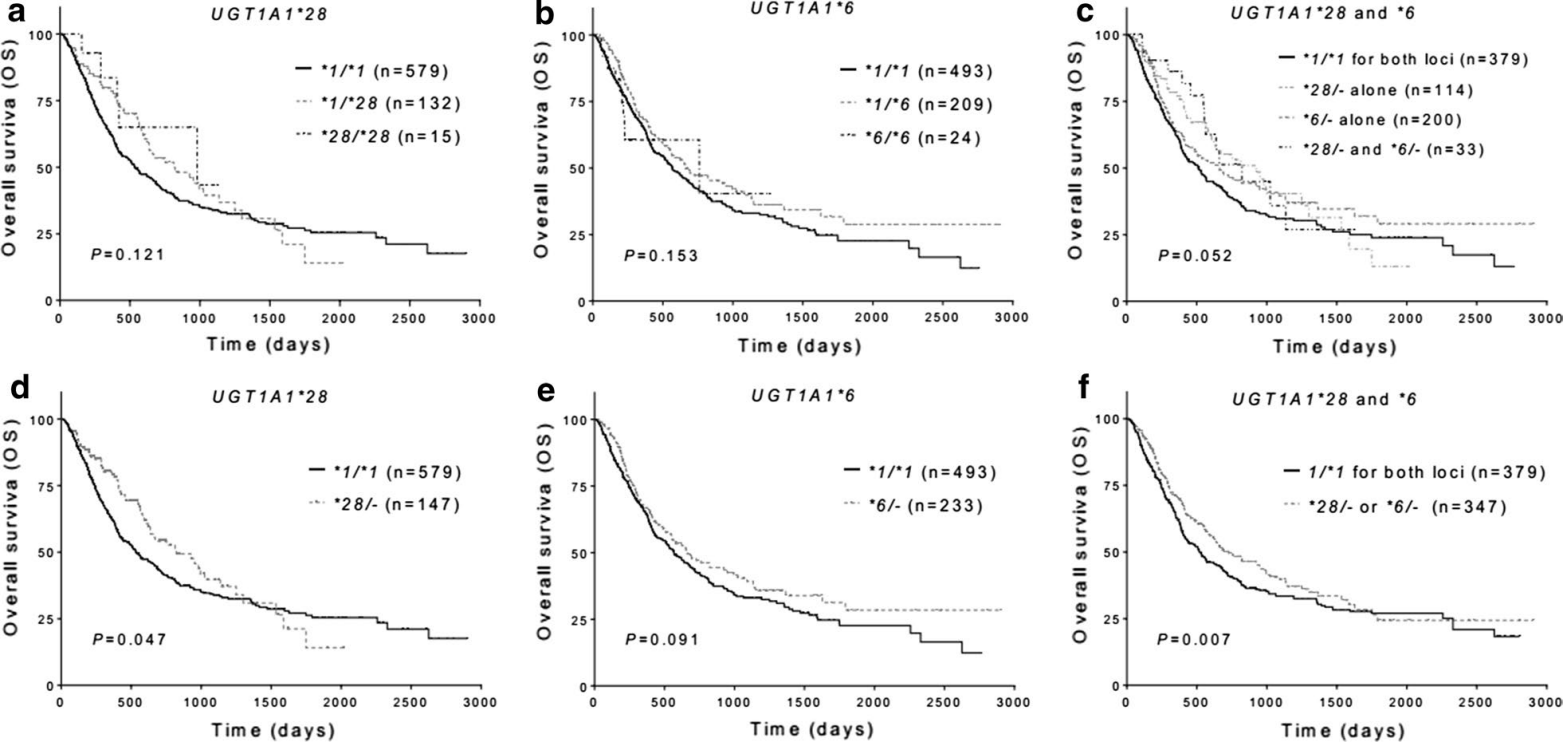
Influence of UGT1A1*28 and *6 polymorphisms on overall survival (OS) in AML patients. **a**, **d** Comparison of OS among genotypes of UGT1A1*28. **b**, **e** Comparison of among genotypes of UGT1A1*6. **c**, **f** Combined effects of UGT1A1*28 and *6 on OS

Association of the *UGT1A1* polymorphisms with OS and EFS of the AML patients was also analyzed. In univariate analysis, patients carrying the UGT1A1*28 allele (*P *= 0.047) or carrying at least one of the UGT1A1*28 and UGT1A1*6 alleles (*P *= 0.007) showed significantly better OS (Fig. 1), carriers of the UGT1A1*6 allele alone showed marginally better OS (*P *= 0.091, Fig. 1e). Median OS was 727 days (range 530–924 day) for carriers of the UGT1A1*28 or UGT1A1*6 alleles, and was 514 days (range 427–601 day) for the UGT1A1*1/*1 homozygotes. In cox proportional hazards model, carrier of at least one of the UGT1A1*28 and UGT1A1*6 alleles showed better OS (HR = 0.787, 95% CI 0.627–0.990, *P *= 0.04), while high risk stratification, WBC count and age were associated with worse OS (Table 4). High risk stratification was also associated with worse EFS (HR = 1.652, 95% CI 1.188–2.297, *P *= 0.003) for the patients. Neither UGT1A1*28 nor UGT1A1*6 polymorphism was associated with EFS of the patients (Additional file 5: Fig. S1).

**Table 4:** *UGT1A1* polymorphisms and clinical factors of Cox regression analysis for OS and EFS

Variables in the model	Overall survival		Event free survival	
	HR (95% CI)	*P* value	HR (95% CI)	*P* value
UGT1A1 *28/− or *6/−	0.787 (0.627–0.990)	0.040	0.959 (0.700–1.313)	0.923
Risk stratification groups:		7.8 × 10^−16^		9.5 × 10^−5^
Low vs intermediate	0.775 (0.566–1.066)	0.117	0.686 (0.493–0.953)	0.021
High vs intermediate	2.615 (2.024–3.377)	1.9 × 10^−13^	1.652 (1.188–2.297)	0.003
WBC count, × 10^9^/l	1.003 (1.001–1.005)	0.002	1.002 (1.000–1.004)	0.095
Age (years)	1.013 (1.005–1.021)	0.002	1.004 (0.995–1.013)	0.366

*WBC* white blood cell

## Discussion

UGT1A is a newly identified enzyme subfamily that is involved in Ara-C detoxification. In this study, we performed an association study on *UGT1A1* polymorphisms with responses to Ara-C and disease prognosis in AML patients for the first time. We observed that carriers of the UGT1A1*6 variant or carriers of any of the UGT1A1*6 and UGT1A1*28 alleles showed significantly decreased risk of non-CR after one and two courses of Ara-C based induction chemotherapy for AML patients. We also observed that carriers of the UGT1A1*6 or UGT1A1*28 alleles showed significantly better OS in AML patients.

Glucuronidation is an important way of metabolism for many small endogenous and exogenous lipophilic compounds and is mediated by UDP-glucuronosyltransferases (UGTs) located in the endoplasmic reticulum. UGT1A1 is the most abundant member of the UGT1A family in human liver and is also the major isoform responsible for the glucuronidation of bilirubin (UGT1A1 specific), SN-38 (the active metabolite of irinotecan), β-estradiol, etc. [33, 34]. UGT1A1*6 and *28 are two functional polymorphisms that lead to decreased glucuronidation activity. UGT1A1*28 is characterized by an extra TA repeat (TA-7) in *UGT1A1* promoter that decreases the gene transcription, and SN-38 glucuronidating activity was decreased by approximately 25 and 50%, respectively, in liver microsomes from UGT1A1*28 heterozygotes and homozygotes [35]. Similarly, UGT1A1*6 is a missense variant that results in decreased UGT1A1 activity by about 50% as indicated by bilirubin and SN-38 glucuronidation [36, 37], and the variant is primarily observed in the Asians. Clinical studies have shown that the two polymorphisms were associated with increased risk of Gilbert’s syndrome or irinotecan-reduced toxicity in Caucasians and Asians [38].

A previous study by Zahreddine reported for the first time that Ara-C is a substrate of UGT1A enzymes and could be inactivated through glucuronidation [20]. Increased UGT1A expression in Ara-C resistant M5 AML THP-1 cells and relapsed AML after standard Ara-C therapies were observed, and elevation in GLI1 expression is sufficient to drive UGT1A dependent glucuronidation of Ara-C and drug resistance [20]. However, by analyzing the mRNA expression profile of the Cancer Genome Atlas (TCGA) dataset, we observed that the expression of *UGT1A1* in blast cells from AML patients was nearly negligible in contrast to the main Ara-C inactivating enzyme *CDA* (Additional file 5: Fig. S3) [39]. These findings suggest that the observed influence of UGT1A1*28 and *6 polymorphisms on Ara-C response in AML patients is less likely to be explained by decreased Ara-C detoxification in AML blast cells. As the UGT1A subfamily is mainly expressed in human liver, we speculate that the UGT1A1*28 and *6 polymorphisms may improve Ara-C response and OS of the AML patients through decreasing hepatic glucuronidation and increasing the systemic exposure of Ara-C. It’s a pity that we failed to detect the plasma concentrations of Ara-C during Ara-C infusion. Of note, AML is usually treated by combined therapy with Ara-C and anthracyclines, and whether UGT1A1 is involved in the metabolism of anthracyclines remains unknown. Therefore, we could not rule out the possibility that difference in disease outcomes among *UGT1A1* genotypes is due to difference in anthracycline metabolism. Influence of both *UGT1A1* polymorphisms on pharmacokinetics and systemic exposure of Ara-C and glucuronidated Ara-C (AraC-Glu) deserved further study in future.

We noticed that the predictive value of UGT1A1*28 and *6 polymorphisms on CR after Ara-C based induction therapy is modest in AML patients (CR rate 76.9% in carriers of the UGT1A1*28 and *6 alleles and 63.8% in UGT1A1*1/*1 for both loci), and the association of UGT1A1*28 alone with non-CR risk was nonsignificant after Bonferroni correction for two SNPs (significance set at *P *< 0.05/2 for 2 SNPs). This may be explained by two reasons: firstly, the lower allele frequency of the UGT1A1*28 polymorphism in our patient cohort. Secondly, the multigenic characteristic of drug response including Ara-C. In our previous studies, we observed that polymorphisms in other genes encoding enzymes in the Ara-C metabolic pathway such as *DCK*, *NME2* (DNPK-B), *RRM2*, and *SAMHD1* are also associated drug response to Ara-C based therapies in AML [10, 16]. We suggest that the construction of a decision tree based on multiple genetic variations concomitantly may increase the predictive values of pharmacogenetics biomarkers in AML induction therapy. Of course, the exact usefulness requires to be explored with a large sample size.

Regarding disease prognosis including OS and EFS, we observed better OS in carriers of UGT1A1*28, or carriers of at least one of the UGT1A1*6 and *28 alleles. Neither polymorphisms nor the combined genotypes were associated with EFS in our study. As EFS was considered only in patients achieved CR after induction therapies, lack of association between *UGT1A1* polymorphisms and EFS observed in our study suggests other mechanisms other than chemosensitivity might also play a role in AML prognosis.

## Conclusions

In summary, we identified *UGT1A1* functional variants as independent factors for better response to Ara-C based chemotherapy and disease prognosis in Chinese AML patients. These findings provide new insightful information for individualized chemotherapy for AML patients. Consideration the ethnic difference in allele frequencies of the *UGT1A* polymorphisms, the positive findings in our study deserved further validation in AML patient cohorts with different ethnic background. Future prospective studies to evaluate influence of *UGT1A1* genotypes on pharmacokinetics and metabolism of Ara-C are also warranted to explore the exact mechanisms of our clinical findings.

## Additional files

**Additional file 1: Table S1.** Primer sequences used to genotype UGT1A1*28 and *6.

**Additional file 2: Table S2.** Clinical features of AML patients according to *UGT1A1* genotypes (additional).

**Additional file 3: Table S3.** Comparison of CR rate among *UGT1A1* genotypes after the first course of induction therapy.

**Additional file 4: Table S4.** Comparison of TRM among *UGT1A1* genotypes after two cycles of induction therapy in AML patients.

**Additional file 5: Fig. S1.** Impact of UGT1A1*28 or *6 on event-free survival (EFS) in AML patients. (a, d) comparison of EFS among genotypes of UGT1A1*28. (b, e) comparison of among genotypes of UGT1A1*6. (c, f) combined effects of UGT1A1*28 and *6 on EFS. **Fig. S2.** Flow chart of the study population. **Fig. S3.** Gene expression of *CDA* and *UGT1A1* mRNA in blasts from AML patients from the Cancer Genome Atlas (TCGA) dataset (n = 173). AML blast cells scarcely express *UGT1A1.*

## Abbreviations

UGT1A1: UDP-glucuronosyltransferase family 1 member A1
UGT1A: UDP-glucuronosyltransferase 1A subfamily
Ara-C: cytarabine
AML: acute myeloid leukemia
CR: complete remission
OR: odds ratio
CI: confidence interval
OS: overall survival
HR: hazard ratio
WBC: white blood cell
*FLT3*: Fms-like tyrosine kinase 3
*NPM1*: nucleophosmin 1
*CEBPA*: CCAAT/enhancer binding protein alpha
*KIT*: KIT proto-oncogene receptor tyrosine kinase
*TP53*: tumor protein p53
*DNMT3a*: DNA methyltransferase 3 alpha
Ara-CTP: cytarabine triphosphate
DCK: deoxycytidine kinase
CDA: cytidine deaminase
SAMHD1: SAM domain and HD domain-containing protein 1
*NME2*: nucleoside diphosphate kinase 2
*RRM2*: ribonucleotide reductase catalytic subunit M2
HH: Hedgehog
GLI: Glioma-associated Oncogene Homolog
MDS: myelodysplastic syndromes
SNPs: single nucleotide polymorphisms
TRM: treatment-related mortality
EFS: event-free survival
T-AML: therapy-related AML
G-CSF: granulocyte colony-stimulating factor
HSCT: hematopoietic stem cell transplantation
PCR: polymerase chain reaction
TCGA: Cancer Genome Atlas
FAB: French–Britain–American
HWE: Hardy–Weinberg Equilibrium
LD: linkage disequilibrium
UGTs: UDP-glucuronosyltransferases
LDH: lactate dehydrogenase
RBC: red blood cell
AraC-Glu: glucuronidated Ara-C
AA: aclacinomycin and cytarabine
DA: daunorubicin and cytarabine
IA: idarubicin and cytarabine
MA: mitoxantrone and cytarabine
TA: pirabucin and cytarabine
