# Supplementary material for: Influence of UGT1A1 polymorphisms on the outcome of acute myeloid leukemia patients treated with cytarabine-base regimens
Source: J Transl Med. 2018 Jul 17;16:197. doi: 10.1186/s12967-018-1579-3 (PMC6050722; doi:10.1186/s12967-018-1579-3)
Supplement: Supplementary file 1 — Additional file 1: Table S1. Primer sequences used to genotype UGT1A1*28 and *6. [file 12967_2018_1579_MOESM1_ESM.docx]

**Table S1.** Primer sequences used to genotype *UGT1A1*28* and **6*

| **Reference SNP ID** | **Forward primers (5’-3’)** | **Reverse primers (5’-3’)** | **Pyrosequencing Primer (5’-3’)** |
| --- | --- | --- | --- |
| *rs8175347* (*UGT1A1*28*) | Biotin-CCCTGCTACCTTTGTGGACTGA | CATGGCGCCTTTGCTCCT | GTTCGCCCTCTCCTACTTATAT |
| *rs4148323* (*UGT1A1*6*) | Biotin-CACCTGACGCCTCGTTGTA | GGAATGGCACAGGGTACGT | TCAAGGTGTAAAATGCTC |
