# Supplementary material for: Influence of UGT1A1 polymorphisms on the outcome of acute myeloid leukemia patients treated with cytarabine-base regimens
Source: J Transl Med. 2018 Jul 17;16:197. doi: 10.1186/s12967-018-1579-3 (PMC6050722; doi:10.1186/s12967-018-1579-3)
Supplement: Supplementary file 2 — Additional file 2: Table S2. Clinical features of AML patients according to UGT1A1 genotypes (additional). [file 12967_2018_1579_MOESM2_ESM.docx]

**Table S2.** Clinical features of AML patients according to *UGT1A1* genotypes (supplementary).

| **Clinical features** | **Total**  **(n=726)** | ***UGT1A1*28*** | | |  | ***UGT1A1*6*** | | |
| --- | --- | --- | --- | --- | --- | --- | --- | --- |
|  |  | **1/*1* (n = 579) | **28/-* (n = 147) | *P* |  | **1/*1* (n = 493) | **6/-* (n = 233) | *P* |
| FLT3-ITD, n (%) |  |  |  |  |  |  |  |  |
| Positive | 78 (10.7) | 58 (10.0) | 20 (13.6) | 0.407 |  | 55 (11.2) | 23 (9.9) | 0.475 |
| Negative | 525 (72.3) | 424 (73.2) | 101 (68.7) |  |  | 360 (73.0) | 165 (70.8) |  |
| Unknown | 123 (16.9) | 97 (17.7) | 26 (16.8) |  |  | 78 (15.8) | 45 (19.3) |  |
| Karyotype |  |  |  |  |  |  |  |  |
| inv(16) or t(16;16) | 22 (3.0) | 16 (2.8) | 6 (4.1) | 0.096 |  | 14 (2.8) | 8 (3.4) | 0.181 |
| t(8;21) | 83 (11.4) | 68 (11.7) | 15 (10.2) |  |  | 49 (9.9) | 34 (14.6) |  |
| Normal cytogenetics | 383 (52.8) | 300 (51.8) | 83 (56.5) |  |  | 261 (52.9) | 122 (52.4) |  |
| +8 alone or t(9;11) | 16 (2.2) | 11 (1.9) | 5 (3.4) |  |  | 10 (2.0) | 6 (2.6) |  |
| Other non-defined | 58 (8.0) | 49 (8.5) | 9 (6.1) |  |  | 45 (9.1) | 13 (5.6) |  |
| Complex (3 or more abnormalities) | 43 (5.9) | 36 (6.2) | 7 (4.8) |  |  | 35 (7.1) | 8 (3.4) |  |
| -5, 5q-, -7, 7q- | 12 (1.7) | 12 (2.1) | - |  |  | 9 (1.8) | 3 (1.3) |  |
| 11q23-non t(9;11) | 8 (1.1) | 8 (1.4) | - |  |  | 7 (1.4) | 1 (0.4) |  |
| t(3;3), t(6;9), t(9;22) | 10 (1.4) | 6 (1.0) | 4 (2.7) |  |  | 6 (1.2) | 4 (1.7) |  |
| Unknown | 91(12.5) | 73 (12.6) | 18 (12.2) |  |  | 57 (11.6) | 34 (14.6) |  |
| First induction therapy regimens n(%) |  |  |  |  |  |  |  |  |
| AA | 151 (20.8) | 126 (21.8) | 25 (17.0) | 0.712 |  | 93 (18.9) | 58 (24.9) | 0.477 |
| DA | 78 (10.7) | 61 (10.5) | 17 (11.6) |  |  | 52 (10.5) | 26 (11.2) |  |
| IA | 157 (21.6) | 121 (20.9) | 36 (24.5) |  |  | 106 (21.5) | 51 (21.9) |  |
| MA | 243 (33.5) | 195 (33.7) | 48 (32.7) |  |  | 173 (35.1) | 70 (30.0) |  |
| TA | 67 (9.2) | 54 (9.3) | 13 (8.8) |  |  | 48 (9.7) | 19 (8.2) |  |
| Other regimens | 30 (4.1) | 22 (3.8) | 8 (5.4) |  |  | 21 (4.3) | 9 (3.9) |  |
| Concomitant drugs |  |  |  |  |  |  |  |  |
| With G-CSF | 137 (18.9) | 112 (17.0) | 25 (19.3) | 0.518 |  | 84 (17.0) | 53 (22.7) | 0.066 |
| With Decitabine | 34 (4.7) | 29 (5.0) | 5 (3.4) | 0.410 |  | 22 (4.5) | 12 (5.2) | 0.682 |

AA, Aclacinomycin and cytarabine; DA, Daunorubicin and cytarabine; G-CSF, Granulocyte Colony-Stimulating Factor; IA, Idarubicin and cytarabine; MA, Mitoxantrone and cytarabine; TA, Pirabucin and cytarabine;

**28/-* or **6/-* represents mutant homozygotes+heterozygotes for corresponding polymorphism.

*P* value based on χ^2^ test for categorical variables.
