# Supplementary material for: Influence of UGT1A1 polymorphisms on the outcome of acute myeloid leukemia patients treated with cytarabine-base regimens
Source: J Transl Med. 2018 Jul 17;16:197. doi: 10.1186/s12967-018-1579-3 (PMC6050722; doi:10.1186/s12967-018-1579-3)
Supplement: Supplementary file 3 — Additional file 3: Table S3. Comparison of CR rate among UGT1A1 genotypes after the first course of induction therapy. [file 12967_2018_1579_MOESM3_ESM.docx]

**Table S3.** Comparison of CR rate among *UGT1A1* genotypes after the first course of induction therapy.

| **SNP** | **Genotype** | **Total (n)** | **CR, n (%)** | **non-CR, n (%)** | **OR (95%CI)** | ***P* value** |
| --- | --- | --- | --- | --- | --- | --- |
| *UGT1A1*6* | **1/*1* | 485 | 182 (37.5) | 303 (62.5) | 1.00 (reference) |  |
|  | **1/*6* | 207 | 90 (43.5) | 117 (56.5) | 0.781 (0.561-1.087) | 0.142 |
|  | **6/*6* | 24 | 16 (66.7) | 8 (33.3) | 0.300 (0.126-0.716) | 0.004 |
|  | **6/-* | 231 | 106 (45.9) | 125 (54.1) | 0.708 (0.516-0.973) | 0.033 |
| *UGT1A1*28* | **1/*1* | 571 | 224 (39.2) | 347 (60.8) | 1.00 (reference) |  |
|  | **1/*28* | 131 | 61 (46.6) | 70 (53.4) | 0.741 (0.505-1.086) | 0.124 |
|  | **28/*28* | 14 | 3 (21.4) | 11 (78.6) | 2.367 (0.653-8.578) | 0.190 |
|  | **28/-* | 145 | 64 (44.1) | 81 (55.9) | 0.817 (0.565-1.181) | 0.282 |
| Combined genotypes | **1/*1* for both loci | 373 | 133 (35.7) | 240 (64.3) | 1.00 (reference) |  |
|  | **6/-* alone | 198 | 91 (46.0) | 107 (54.0) | 0.652 (0.459-0.925) | 0.017 |
|  | **28/-* alone | 112 | 49 (43.8) | 63 (56.3) | 0.713 (0.464-1.094) | 0.122 |
|  | **6/-* and **28/-* | 33 | 15 (45.5) | 18 (54.5) | 0.665 (0.325-1.362) | 0.265 |
|  | **6/-* or **28/-* | 343 | 155 (45.2) | 188 (54.8) | 0.672 (0.498-0.907) | 0.009 |
