# Supplementary material for: Influence of UGT1A1 polymorphisms on the outcome of acute myeloid leukemia patients treated with cytarabine-base regimens
Source: J Transl Med. 2018 Jul 17;16:197. doi: 10.1186/s12967-018-1579-3 (PMC6050722; doi:10.1186/s12967-018-1579-3)
Supplement: Supplementary file 4 — Additional file 4: Table S4. Comparison of TRM among UGT1A1 genotypes after two cycles of induction therapy in AML patients. [file 12967_2018_1579_MOESM4_ESM.docx]

**Table S4.** Comparison of TRM among *UGT1A1* genotypes after two cycles of induction therapy in AML patients.

| **SNP** | **Genotype** | **Total (n)** | **TRM, n (%)** | **No TRM, n (%)** | **OR (95%CI)** | ***P* value** |
| --- | --- | --- | --- | --- | --- | --- |
| *UGT1A1*28* | **1/*1* | 553 | 29 (5.2) | 524 (94.8) | 1.00 (reference) |  |
|  | **1/*28* | 130 | 7 (5.4) | 123 (94.6) | 1.028 (0.440-2.402) | 0.949 |
|  | **28/*28* | 14 | - | 14 (100.0) | - | 1.000^†^ |
|  | **28/-* | 144 | 7 (4.9) | 137 (95.1) | 0.923 (0.396-2.153) | 0.853 |
| *UGT1A1*6* | **1/*1* | 471 | 29 (6.2) | 442 (93.8) | 1.00 (reference) |  |
|  | **1/*6* | 202 | 6 (3.0) | 196 (97.0) | 0.467 (0.191-1.142) | 0.088 |
|  | **6/*6* | 24 | 1 (4.2) | 23 (95.8) | 0.663 (0.086-5.081) | 0.690 |
|  | **6/-* | 226 | 7 (3.1) | 219 (96.9) | 0.487 (0.210-1.130) | 0.088 |

TRM, treatment-related mortality.

^†^*P* value is based on Fisher's exact test.
