# Supplementary material for: Influence of UGT1A1 polymorphisms on the outcome of acute myeloid leukemia patients treated with cytarabine-base regimens
Source: J Transl Med. 2018 Jul 17;16:197. doi: 10.1186/s12967-018-1579-3 (PMC6050722; doi:10.1186/s12967-018-1579-3)

**Figure legends (additional)**

**Figure S1.** Impact of *UGT1A1*28* or **6* on event-free survival (EFS) in AML patients. (a, d) comparison of EFS among genotypes of *UGT1A1*28*. (b, e) comparison of among genotypes of *UGT1A1*6*. (c, f) combined effects of *UGT1A1*28* and *6 on EFS.


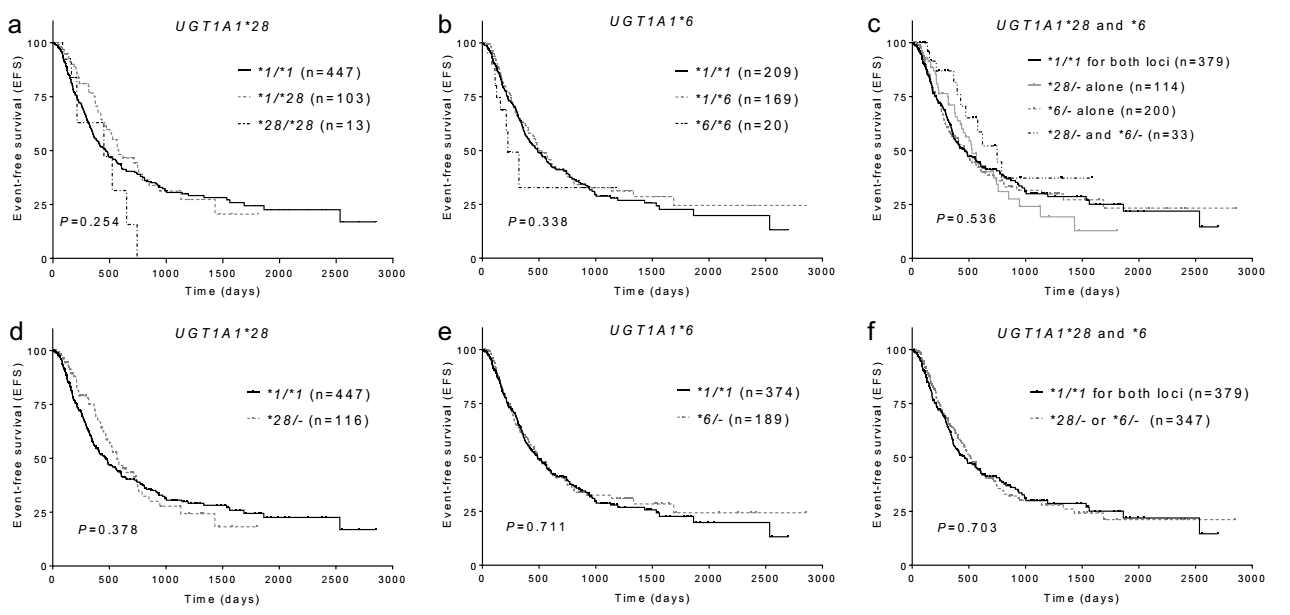


**Figure S2.** Flow chart of the study population.


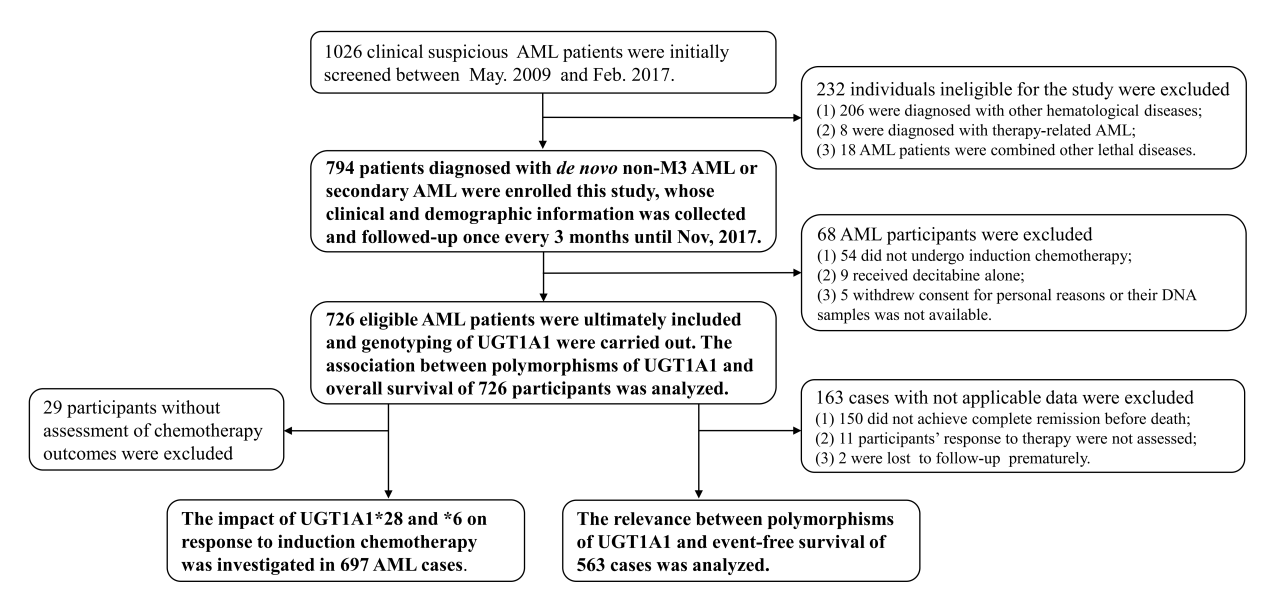


**Figure S3.** Gene expression of *CDA* and *UGT1A1* mRNA in blasts from AML patients from the Cancer Genome Atlas (TCGA) dataset (n=173). AML blast cells scarcely express *UGT1A1*.


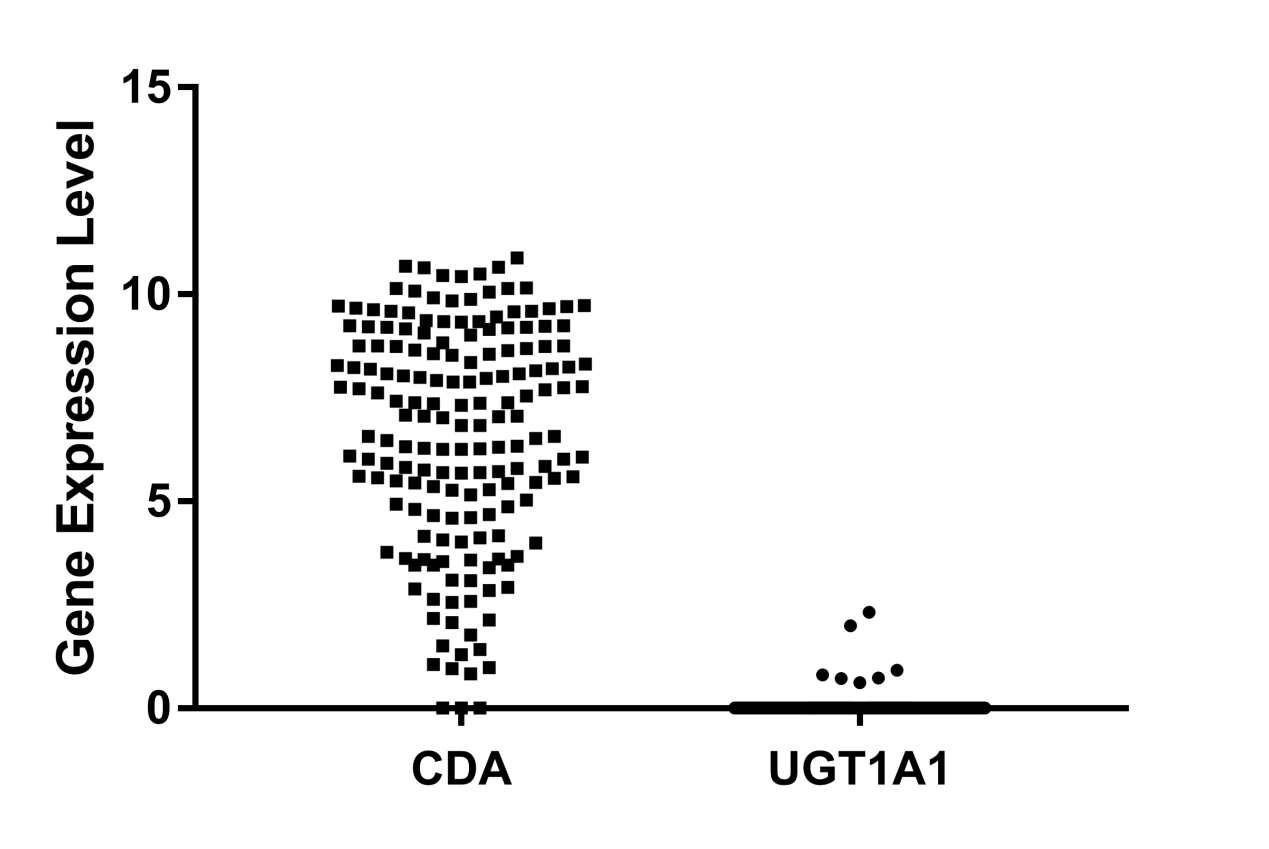

Supplement: Supplementary file 5 — Additional file 5: Fig. S1. Impact of UGT1A1*28 or *6 on event-free survival (EFS) in AML patients. (a, d) comparison of EFS among genotypes of UGT1A1*28. (b, e) comparison of among genotypes of UGT1A1*6. (c, f) combined effects of UGT1A1*28 and *6 on EFS. Fig. S2. Flow chart of the study population. Fig. S3. Gene expression of CDA and UGT1A1 mRNA in blasts from AML patients from the Cancer Genome Atlas (TCGA) dataset (n = 173). AML blast cells scarcely express UGT1A1. [file 12967_2018_1579_MOESM5_ESM.docx]
